# Molecular Epidemiology, Antibiotic Resistance, and Virulence Traits of *Stenotrophomonas maltophilia* Strains Associated With an Outbreak in a Mexican Tertiary Care Hospital

**DOI:** 10.3389/fcimb.2020.00050

**Published:** 2020-02-18

**Authors:** Ariadnna Cruz-Córdova, Jetsi Mancilla-Rojano, Víctor M. Luna-Pineda, Gerardo Escalona-Venegas, Vicenta Cázares-Domínguez, Christopher Ormsby, Isabel Franco-Hernández, Sergio Zavala-Vega, Mónica Andrés Hernández, Marisol Medina-Pelcastre, Israel Parra-Ortega, Daniela De la Rosa-Zamboni, Sara A. Ochoa, Juan Xicohtencatl-Cortes

**Affiliations:** ^1^Laboratorio de Investigación en Bacteriología Intestinal, Hospital Infantil de México “Federico Gómez”, Ciudad de México, Mexico; ^2^Centro de Investigación en Enfermedades Infecciosas, Instituto Nacional de Enfermedades Respiratorias, Ciudad de México, Mexico; ^3^Laboratorio Central de Bacteriología, Hospital Infantil de México “Federico Gómez”, Ciudad de México, Mexico; ^4^Laboratorio de Neuropatología, Instituto Nacional de Neurología y Neurocirugía “Manuel Velasco Suárez”, Ciudad de México, Mexico; ^5^Hospital Ángeles Morelia, Morelia, Mexico; ^6^Departamento de Epidemiología, Hospital Infantil de México “Federico Gómez”, Ciudad de México, Mexico

**Keywords:** outbreak, *S. maltophilia*, molecular typing, virulence, resistance

## Abstract

*Stenotrophomonas maltophilia*, an emerging opportunistic pathogen, is widely distributed in the environment the resistance mechanisms, and virulence factors of this bacterium facilitate its dissemination in hospitals. This study aimed to characterize the molecular epidemiology of *S. maltophilia* strains associated with an outbreak in the Children's Hospital of México Federico Gómez (HIMFG). Twenty-one clinical *S. maltophilia* strains were recovered from cultures of blood and urine samples from 10 pediatric patients at the emergency department, and nine environmental *S. maltophilia* strains recovered from faucets in the same area were also included. Two of the 10 patients were related with health care-associated infections (HCAIs), and the other eight patients (8/10) were infected with environmental *S. maltophilia* strains. The outbreak was controlled by monthly disinfection of the faucets in the emergency department. Typing using pulsed-field gel electrophoresis (PFGE) showed a 52% genetic diversity with seven pulsotypes denoted P1–P7 among all *S. maltophilia* strains. Three pulsotypes (P2, P3, and P7) were identified among both the clinical and environmental *S. maltophilia* strains and associated with two type sequences (STs), namely, ST304 and ST24. Moreover, 80% (24/30) of the strains exhibited resistance mainly to tetracycline, 76.66% (23/30) to trimethoprim-sulfamethoxazole, and 23.33% (7/30) to the extended-spectrum β-lactamase (ESBL) phenotype. The main resistance genes identified by multiplex PCR were *sul1* in 100% (30/30), *qnr* in 86.66% (26/30), and *intl1* in 80% (24/30) of the samples, respectively. Furthermore, the *pilU, hlylII*, and *rmlA* genes were identified in 96.6% (29/30), 90% (27/30), and 83.33% (25/30) of the samples, respectively. Additionally, 76.66% (23/30) of the *S. maltophilia* strains exhibited high swimming motility, 46.66% (14/30) showed moderate biofilm formation capacity, 43.33% (13/30) displayed moderate twitching motility, and 20% (6/30) exhibited high adherence. The clinical *S. maltophilia* strains isolated from blood most strongly adhered to HTB-9 cells. In conclusion, the molecular epidemiology and some of the features such as resistance, and virulence genes associated with colonization patterns are pathogenic attributes that can promote *S. maltophilia* dissemination, persistence, and facilitate the outbreak that occurred in the HIMFG. This study supports the need for faucet disinfection as a control strategy for clinical outbreaks.

## Introduction

*Stenotrophomonas maltophilia* is an intrinsically multidrug-resistant (MDR) gram-negative bacillus that is widely distributed in aqueous and humid habitats in the environment, including plants, animals, and water sources (Gulcan et al., 2004). *S. maltophilia* is recognized as an important nosocomial pathogen that causes health care-associated infections (HCAIs) through direct contact, ingestion, aspiration, aerosolization of potable water or the hands of healthcare workers (Guyot et al., 2013). This opportunistic pathogen can cause severe infections, such as bacteremia, sepsis, pneumonia, meningitis after neurosurgical procedures, endocarditis, urinary tract infection, septic arthritis, and endophthalmitis, in immunocompromised patients (Denton and Kerr, 1998; Park et al., 2008; Botana-Rial et al., 2016; Waite et al., 2016). HCAI-associated *S. maltophilia* strains exhibit resistance to a broad spectrum of antibiotics, including β-lactams, macrolides, fluoroquinolones, aminoglycosides, chloramphenicol (C), tetracyclines (TEs), polymyxins, and trimethoprim-sulfamethoxazole (SXT) (Wu et al., 2006; Chang et al., 2007). The intrinsic resistance of *S. maltophilia* strains is associated with low membrane permeability, efflux pumps, and the intrinsic beta-lactamases L1 and L2, among other drug resistance determinants that shield this microorganism (Sanchez et al., 2002; Crossman et al., 2008; Mojica et al., 2019). *S. maltophilia* can also acquire resistance via the acquisition of mutations or resistance genes by horizontal gene transfer (Sanchez, 2015). Among these genes, dihydropteroate synthase (*sul1* and *sul2*), and dihydrofolate reductase (*drfA*) are the main mechanisms of SXT resistance in *S. maltophilia* (Toleman et al., 2007).

Some virulence factors have been described in *S. maltophilia*, including Smf1-fimbrial operon, proteases, hemolysins, exopolysaccharides (EPSs), lipopolysaccharides (LPSs), siderophores, flagella, lipases, putative virulence genes in a pathogenicity island encoding proteins from the RTX (repeats-in-toxin) family, and transmembrane secretion system components (Brooke, 2012; Adamek et al., 2014; Sanchez, 2015; Pompilio et al., 2016; Alcaraz et al., 2018; Trifonova and Strateva, 2019). A few studies have described the colonization of *S. maltophilia* in cultures of HEp-2 and IB3-1 cells as well as A/J, DBA/2, BALB/c, and C57BL/6 mice (De Vidipo et al., 2001; De Oliveira-Garcia et al., 2003; Pompilio et al., 2008; Lin et al., 2015). Outbreaks of *S. maltophilia* strains have been associated with a high clonal diversity by pulsed-field gel electrophoresis (PFGE), and these analyses included investigations of the population structure of bacteria from cystic fibrosis patients (Nicoletti et al., 2011; Vidigal et al., 2014). PFGE has been used to analyze the profiles of the environmental reservoirs of epidemic clones, mainly in damp areas (taps, water reservoirs of humidifiers, assisted ventilation equipment, aspirators, and ventilators), and their modes of transmission (Valdezate et al., 2004; Gherardi et al., 2015). Outbreaks of *S. maltophilia* clinical strains have been associated with intensive care units (ICUs), emergency departments, respiratory units, oncology units, and surgery wards (Brooke, 2012). In this study, the molecular epidemiology of clinical and environmental strains of outbreak-associated *S. maltophilia* was characterized by identifying their virulence, antibiotic resistance, twitching motility, biofilm formation capacity, and adherence profiles. Our results suggest that some of these features can promote *S. maltophilia* dissemination and persistence in the HIMFG.

## Materials and Methods

### Bacterial Strains

A collection of 21 clinical strains of *S. maltophilia* was obtained between December 19th, 2016, and February 13th, 2017 (Table 1). These strains were sourced from the blood and urine of pediatric patients who were accepted into the emergency department and subsequently hospitalized in different wards (surgery, internal medicine, intensive therapy unit, neurology, infectology, neurosurgery, oncology, and surgical therapy). Nine environmental strains of *S. maltophilia* recovered from faucets in the emergency department were also included in this study. *S. maltophilia* strains were routinely cultured in blood agar (5% sheep blood) overnight at 37°C and identified using the MALDI-TOF VITEK MS Microbial Identification System (bioMérieux, 376 Chemin de l'Orme, 69280 Marcy-l'Étoile, France) at the Central Clinical Laboratory of the Children's Hospital of México Federico Gómez (HIMFG) (Tatman-Otkun et al., 2005; Jacquier et al., 2011).

**Table 1:** MICs to six antibiotic classes suggested by the CLSI (2018) obtained for 21 clinical strains and nine environmental strains of *S. maltophilia* associated with a hospital outbreak.

**Type**	**Strains/Date of isolation**	**Sources**	**MIC to different antibiotic classes (μg/mL)**						**ARP**	**PHE-ESBL**	**Pulsotypes (P)**
			**βL/I (TIM)**	**C 3rd (CAZ)**	**FQ (LVX)**	**FPI (SXT)**	**TEs (TE)**	**PH (C)**			
Environment	Env1 (12/29/2016)	Short-stay unit Exit-S	1	0.5	0.062	0.125	0.5	1	S	–	P7
	Env2 (12/29/2016)	Short-stay unit Entrance-F	1	1	0.125	256	32	0.25	R	–	P7
	Env3 (12/29/2016)	Short-stay unit Entrance-S	256	64	0.5	256	32	8	MDR	+	P3
	Env4 (12/29/2016)	Patient observation area S, F	64	64	0.5	256	32	8	MDR	+	P3
	Env5 (12/29/2016)	Areas A and B-S	64	64	256	256	16	256	MDR	–	P2
	Env6 (12/29/2016)	Areas A and B-F	1	2	256	256	32	8	MDR	–	P7
	Env7 (12/29/2016)	Areas C-S	1	1	256	256	32	4	MDR	–	P7
	Env8 (12/29/2016)	Areas D-F	2	1	0.062	256	2	4	R	–	P7
	Env9 (12/29/2016)	Areas D-F	64	16	2	256	16	256	MDR	–	P2
Pat1	490U (01/10/2107)	Urine	0.125	4	0.062	0.031	64	0.031	R	–	P1
	653U (01/10/2017)	Urine	256	256	1	256	64	0.125	MDR	–	P5
	717H (01/20/2017)	Blood	16	1	0.25	256	32	2	R	–	P6
	695H (01/20/2017)	Blood	1	1	0.031	256	4	0.25	R	–	P6
	723H (01/21/2017)	Blood	0.5	2	0.062	256	32	2	R	–	P6
	160U (02/09/2017)	Urine	256	64	8	256	4	64	MDR	+	P4
	178U (02/10/2017)	Urine	256	256	256	256	256	64	PDR	+	P4
Pat2	41H (12/26/2016)	Blood	0.062	1	0.062	0.25	0.25	0.25	S	–	P7
Pat3	488H (01/13/2017)	Blood	2	2	0.125	0.125	32	2	R	–	P7
Pat4	35H (12/26/2016)	Blood	32	2	0.25	256	32	8	MDR	–	P3
Pat5	339H (02/12/2017)	Blood	128	64	0.125	256	32	8	MDR	+	P5
	385H (02/13/2017)	Blood	1	2	0.031	0.031	2	0.25	S	–	P5
	635H (01/19/2017)	Blood	128	32	0.062	8	8	8	MDR	+	P5
	581H (01/18/2017)	Blood	128	32	0.062	0.25	16	8	MDR	+	P5
Pat6	483H (01/13/2017)	Blood	0.5	1	0.125	256	32	8	R	–	P7
Pat7	899H (01/26/2017)	Blood	0.25	1	0.062	256	32	32	MDR	–	P7
Pat8	63H (12/26/2016)	Blood	0.25	1	0.125	256	16	8	R	–	P7
	50H (12/12/2016)	Blood	0.25	1	256	256	32	2	MDR	–	P7
Pat9	499H (01/13/2017)	Blood	0.5	2	0.062	0.25	16	32	R	–	P7
Pat10	931H (12/19/2016)	Blood	64	64	16	256	8	256	PDR	–	P2
	54H (12/26/2016)	Blood	64	64	16	256	16	256	PDR	–	P2
	^*^CVR		≥32	≥16	≥4	≥4	≥8	≥16	–	–	
	^**^TP (%)		43.33	40.0	26.66	76.66	80.00	23.33	–	23.33	

Env1–Env9 are environmental samples, Areas A–B are geographical areas of the emergency department, faucet (F), spout (S), antibiotic resistance profile (ARP), β-lactam/β-lactamase inhibitor combinations [βL/I: ticarcillin-clavulanate (TIM)]; third-generation cephems [C 3rd: ceftazidime (CAZ)]; fluoroquinolones [FQ: levofloxacin (LVX)]; folate pathway inhibitors [FPI: trimethoprim-sulfamethoxazole (SXT)]; tetracyclines [TEs: tetracycline (TE)]; phenicols [PH: chloramphenicol (C)]; S, susceptible to six antibiotic classes; R, resistant to one or two antibiotic classes; MDR, multidrug-resistant, resistant to three or more antibiotic classes; PDR, pandrug-resistant, resistant to six antibiotic classes; phenotypic ESBL (PHE-ESBL).

* Cutoff values for resistance to the MIC (μg/mL; CVR). Pat1-Pat10 are patients.

** *Total percentage [TP (%)]; negative result (–), positive result (+). The shaded boxes indicate the values of resistant MICs. The gray boxes indicate the antibiotics to which each strain shows resistance*.

### Antimicrobial Susceptibility and Extended-Spectrum β-Lactamase (ESBL) Phenotypic Detection in the *S. maltophilia* Strains

Tests of the antimicrobial susceptibility of the *S. maltophilia* strains were performed according to the Clinical and Laboratory Standards Institute (CLSI, 2018). The minimum inhibitory concentration (MIC) was determined through a microdilution test in Muller-Hinton broth (NJ, USA). Several antibiotic categories were included in this study: TEs (TE) from Heritage Pharmaceuticals Inc. (Edison, NJ, USA), β-lactam/β-lactamase inhibitor combinations (ticarcillin/clavulanate, TIM) from Sigma-Aldrich (St. Louis, MO, USA), third-generation cephems (ceftazidime, CAZ) from Roselle Rd (Schaumburg, IL, USA), fluoroquinolones (levofloxacin, LVX) from MP Biomedicals (Solon, OH, USA), folate pathway inhibitors (SXT), and phenicols (C) from Roche (Basel, Switzerland). *Escherichia coli* ATCC 25922 and *Pseudomonas aeruginosa* ATCC 27853 were used as controls.

The extended-spectrum β-lactamase (ESBL) phenotypes were detected via double-disk synergy testing to evaluate the synergistic effects between clavulanic acid and β-lactam antibiotics as previously described by our research group (Ochoa et al., 2016; CLSI, 2018).

### Detection of Quinolone, Sulfonamide Resistance, and Integron-Integrase Gene Classes in the *S. maltophilia* Strains

Genomic DNA from the *S. maltophilia* strains after culture in trypticase soy broth (TSB, Difco-Becton Dickinson, NJ, USA) overnight at 37°C was obtained using the Wizard® Genomic DNA Purification Kit from Promega Corporation (Madison, WI, USA). The obtained genomic DNA was used for identification of the resistance genes (*sul1, sul2*, and *qnr*) through end-point PCR and the integron classes (1, 2, and 3) by multiplex PCR (Table S1) (Ozkaya et al., 2014). The DNA products were resolved by electrophoresis and stained with 0.5 μg/mL ethidium bromide (Sigma-Aldrich, St. Louis, MO, USA; Ochoa et al., 2016).

### Identification of *S. maltophilia* Strain Virulence Genes

DNA was extracted from *S. maltophilia* strains as described in Detection of quinolone, sulfonamide resistance and integron-integrase gene classes in the S. maltophilia strains. The primers used for the identification of 20 virulence genes (*virB, rmlA, fliC, stmPr1, pilU, hlyIII, gspD, papD, afaD, hgbB, entA, tpsB, picN1, motA, zot, fhaB, frpC, fimH, lktD*, and *hcp*) by multiplex PCR were designed based on the following sequences: *S. maltophilia* K279a, *S. maltophilia* R551_3, *S. maltophilia* SJTH1, *S. maltophilia* NCTC10257, *S. maltophilia* FDAARGOS_92, 13637, *S. maltophilia* WZN_1, *S. maltophilia* JV3, *S. maltophilia* CSM2, and *S. maltophilia* ISMMS2 (Table S2). The PCR products were resolved by electrophoresis on agarose gels and stained with 0.5 μg/mL ethidium bromide for visualization as previously described (Ochoa et al., 2016).

### Biofilm and Adherence Assays of the *S. maltophilia* Strains

Twenty microliters (1 × 10^7^ bacteria/mL) of *S. maltophilia* strains cultured overnight in TSB were added to 180 μL of TSB in each well of a 96-well plate and incubated for 24 h at 37°C. The medium was then discarded from each well, and the growing bacteria were removed by washing three times with sterile 1× phosphate-buffered saline (PBS, pH 7.3, Sigma-Aldrich). The biofilms formed by the *S. maltophilia* strains were fixed with 200 μL of 2% formaldehyde, stained with crystal violet for 1 h and washed three times with 1× PBS to remove excess dye. Subsequently, 200 μL of methanol was added to each well to elute the crystal violet, and the absorbance at 620 nm (OD_620_) was quantified using a Multiskan FC instrument from Thermo Scientific (Waltham, MA, USA). The experiments were performed in triplicate at least three times on three different days. The data are presented as the means of the averages of the results obtained from all the experiments. The values for low, moderate and high biofilm formation capacity were determined based on the OD_620_ of the biofilm formed by *S. maltophilia* ATCC 13637 according to the protocol described by Pompilio et al. (2011).

The adherence assays were performed using HTB-9 bladder cells (5637, ATCC® HTB9™; Manassas, VA, USA), which were derived from a human urinary bladder grade II carcinoma. HTB-9 bladder cells were cultured in Roswell Park Memorial Institute medium (RPMI-1640; ATCC®, Manassas, VA, USA) supplemented with 10% fetal bovine serum (ATCC®, Manassas, VA, USA). The HTB-9 cells were counted with trypan blue (ATCC®, Manassas, VA, USA) and the number of bacteria was adjusted to a multiplicity of infection (MOI) of 100:1 (1 × 10^5^ cells/mL/well) in 24-well plates with RPMI-1640. The cell monolayers were infected with 1 × 10^7^ bacteria/mL and incubated for 3 h at 37°C with 5% CO_2_. To remove non-attached bacteria, the supernatant was discarded, and the infected cell monolayers were washed three times with 1× PBS. The bacteria attached to the cell monolayers were removed after adding 1 mL of 0.1% Triton X-100 in PBS (Amresco, OH, USA), and serial dilutions were plated onto Luria-Bertani (LB) agar plates to determine the number of colony-forming units (CFU)/mL. The adherence assays were performed in triplicate three times on different days, and the CFU/mL values are expressed as the means of the averages. The environmental and clinical *S. maltophilia* strains were considered to adhere at low, moderate or high levels based on the adherence value (CFU/mL) of the *S. maltophilia* ATCC 13637 control strains, and the standard deviation of the events was recorded (De Oliveira-Garcia et al., 2003).

### PFGE and MLST Analyses of the *S. maltophilia* Strains

*S. maltophilia* strains were analyzed by PFGE according to methods modified by our research group (Ochoa et al., 2016). Briefly, purified DNA was digested with 2 U of *Xba*l (Invitrogen, Life Technologies, Waltham, MA, USA) at 37°C for 4 h. The DNA samples were electrophoresed on a CHEF Mapper system from Bio-Rad Life Science Research (Hercules, CA, USA) for 23 h at 200 V (6 V/cm). The macro restriction products were resolved by electrophoresis on 1.2% agarose gels, stained with 0.5 μg/mL ethidium bromide for 40 min and visualized under UV light using a ChemiDoc MP® imaging system. The PFGE patterns were analyzed using NTSYS-pc software, and the clonality degree was estimated according to the criteria described by Tenover et al. (1995).

MLST of *S. maltophilia* strains were also performed following the protocol described by Kaiser et al. (2009), Jolley et al. (2018). The seven housekeeping genes (*atpD, gapA, guaA, mutM, nuoD, ppsA*, and *recA*) were amplified using Phusion TM High-fidelity DNA polymerase (Thermo Fisher, Waltham, MA, USA), purified and then after sequenced using the BigDye Terminator v3.1 Cycle Sequencing Kit (Applied Biosystems, Foster City, CA, USA) with an ABI 3500 Genetic Analyzer (Applied Biosystems) automatic sequencer. The allelic profiles of each gene were analyzed using Chromas v2.6.6 (http://technelysium.com.au/wp/chromas-version-history/) and BioEdit v7.0.5.3 (https://www.nucleics.com/DNA_sequencing_support/Trace_viewer_reviews/BioEdit/) software, and the ST for each unique allelic profile was designated based on information from the MLST website (https://pubmlst.org/smaltophilia/). In addition, the primer sequences for the *gapA, mutM, nuoD*, and *ppsA* housekeeping genes were manually designed. The allele sequences of these genes were obtained from the *S. maltophilia* MLST database (Oxford). Multiple sequence alignment was performed by identifying conserved regions. The designed MLST primers generated products of 768 bp for *gapA*, 592 bp for *mutM*, 531 bp for *nuoD*, and 887 bp for *ppsA* (Table S1).

### Twitching and Swimming Motility Assays

For the assessment of twitching motility, the *S. maltophilia* strains were first grown on TSB at 37°C for 24 h, and 1 μL of each bacterial suspension (1.5 × 10^8^ bacteria/mL) was then vertically inoculated into a well of a six-well plate with 5 mL of TSB and 1% agar and incubated at 37°C for 24 h. The colonies exhibiting twitching motility were then visualized by staining with Coomassie Brilliant Blue R-250 (Bio-Rad, Hercules, USA).

To assess the swimming motility, the *S. maltophilia* strains were grown overnight in TSB at 37°C, and 1 μL of each bacterial suspension (1.5 × 10^8^ bacteria/mL) was vertically inoculated into a well of a six-well plate containing 5 mL of TSB and 0.3% agar and incubated at 37°C for 24 h.

The twitching and swimming motilities were evaluated by measuring the halo of growth in millimeters (mm) around the inoculation zone, and one, two and four standard deviations indicated low, moderate, and high profiles, respectively (Pompilio et al., 2011). The strains *P. aeruginosa* ATCC 27853, *E*. *coli* ATCC 25922, and *S. maltophilia* ATCC 13637 were used as controls in both experiments.

### Statistical Analysis

ANOVA followed by Fisher's protected least significant difference (PLSD) was used to identify significant differences in the data, and values of *p* < 0.05 were considered to indicate statistical significance. The PFGE results were analyzed with NTSYS-pc software (version 2.0, Applied Biostatistics, Inc., NY, USA) based on the simple matching similarity coefficient and the unweighted pair group method using arithmetic averages (UPGMA). The clonal complexes were analyzed using PHYLOViZ software (https://online.phyloviz.net/index), which is based on the goeBURST algorithm (Francisco et al., 2009). The biofilm, adherence, twitching, and swimming motility data were analyzed with StatView for Windows software (www.statview.com) (Landau and Rabe-Hesketh, 1999). The heat maps were generated using GraphPad Prism v8.0 software (https://www.graphpad.com/). The association among the clonal, clinical, and environmental *S. maltophilia* strains was analyzed using ShinyCircos software (https://github.com/venyao/shinyCircos) (Yu et al., 2018).

## Results

### Outbreak Description

**Figure 1:**
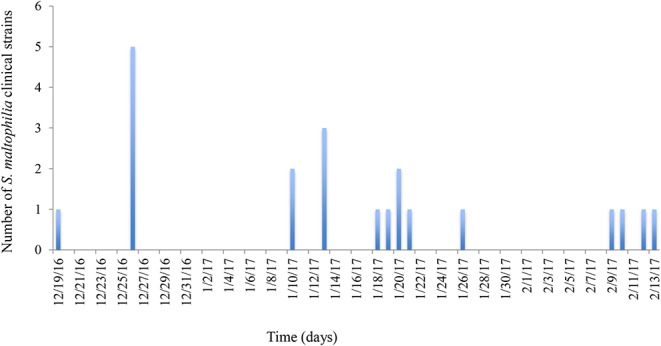
Graphical representation of the epidemiology of outbreak-associated *S. maltophilia* strains over time. The frequencies of clinical strains were determined by their persistence time during the outbreak. The first strain was identified on December 19th, 2016, and the last strain was identified on February 13th, 2017. The culture of environmental *S. maltophilia* strains was performed on December 26, 2016.

The HIMFG is a third-level hospital that sees pediatric patients, and the decision of whether to admit patients occurs in the emergency department. The HCAI rate is between 0 and 4%, and the incidence is between 0 and 4.2/1,000 patients each day in the emergency department. A total of 30 *S. maltophilia* strains were identified as follows: 17 from blood (December 19th, 2016–February 13th, 2017), four from urine (January 10th, 2017–February 13th, 2017), and nine from the environment (December 29th, 2016; Figure 1, Table 1). The cultures from blood and urine were recovered from 10 patients in the emergency department.

To study the outbreak, bacterial samples were obtained from surfaces of the nursery trolley, beds of patients (handrails and mattresses), aseptic material (single-use chlorhexidine and alcohol devices), storage drawers, water faucets, and the surrounding and interior regions of the spouts and cultured in blood culture bottles. Only the water faucets and spout cultures were positive for *S. maltophilia* strains. As control measures, the sampling technique was reviewed at the beginning of the outbreak, and blood cultures and hand hygiene were emphasized (De la Rosa-Zamboni et al., 2018). After *S. maltophilia* strains were found in the external and internal parts of the faucets, the faucets were dismantled, cleaned, disinfected with chlorine (400 ppm) and sterilized in an autoclave. A faucet disinfection policy was subsequently implemented in the emergency department, and this policy involved cleaning and disinfection every 2 weeks for the first 3 months after the outbreak and then every month thereafter, which resulted in the eradication of *Stenotrophomonas* infections in the emergency department.

### Genetic Diversity of the *S. maltophilia* Strains by PFGE and MLST Analyses

**Figure 2:**
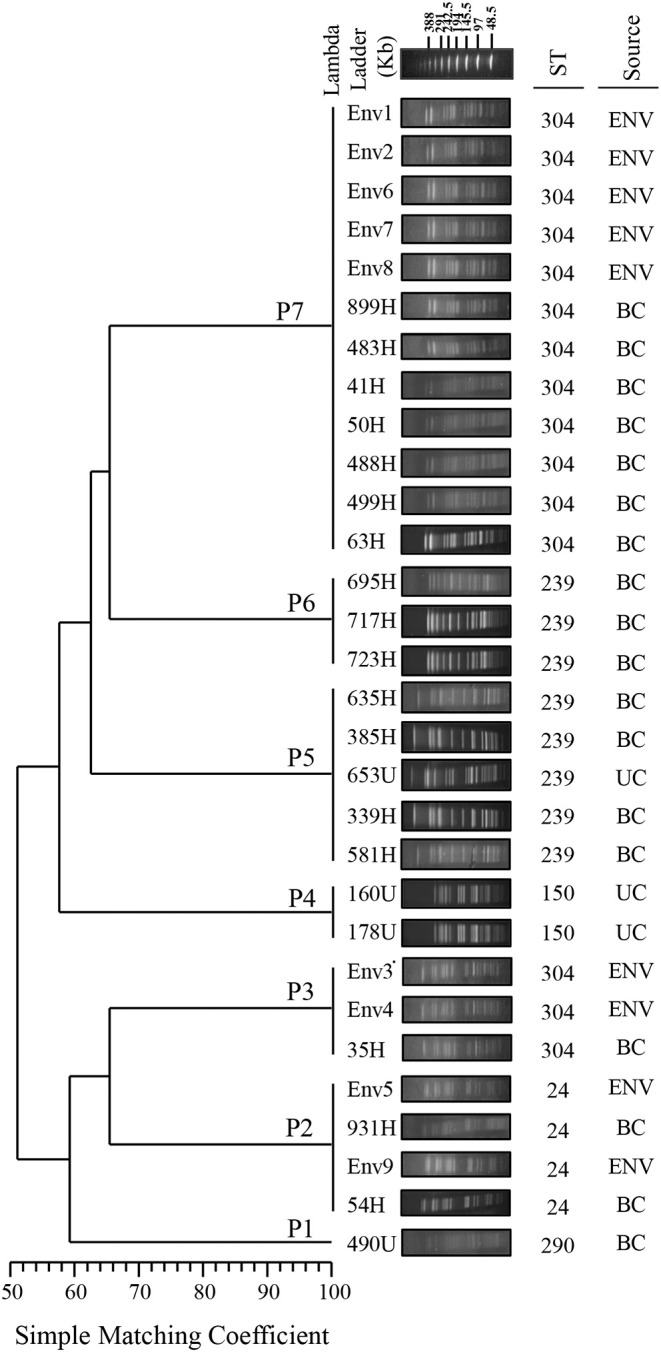
Dendrogram of the PFGE pulsotypes in 21 clinical strains and nine environmental strains of *S. maltophilia*. The diversity analysis was performed using NTSYS-pc software (version 2.0, Applied Biostatistics, Inc., NY, USA). The absence/presence matrix was evaluated based on the simple matching coefficient using the UPGMA algorithm. The dendrogram was evaluated using the cophenetic correlation coefficient obtained with the Mantel test, which indicated the dispersion of the data and showed a value of *r* = 0.98319. The diversity clusters and associated pulsotypes (P) are indicated, and the sequence type (ST) determined by MLST and the source are shown. Environmental (ENV), blood culture (BC) and urine culture (UC).

The PFGE analysis revealed that the clinical and environmental strains of *S. maltophilia* clustered in seven pulsotypes (P1–P7) carrying between 17 and 21 DNA fragments, and the molecular weights ranged from <48.5 to >338 kb. A genetic diversity analysis of each *S. maltophilia* strain was performed with NTSYS-pc software to obtain a matrix of the absence/presence of the identified bands. The simple matching coefficient (Sokal and Michener, 1958) and UPGMA organization allowed the grouping of the *S. maltophilia* strains into seven pulsotypes, which exhibited 52% similarity among themselves (Figure 2). The cophenetic correlation coefficient obtained from the dendrogram using the Mantel test indicated the dispersion of the data and found a value of *r* = 0.98319. The seven pulsotypes showed the following distributions: 3.33% (1/30) of the *S. maltophilia* strains belonged to pulsotype P1 (strain 490U), 13.33% (4/30) belonged to pulsotype P2 (strains 54H, 931H, Env5, and Env6), 10.00% (3/30) belonged to pulsotype P3 (strains 35H, Env3, and Env4), 6.66% (2/30) belonged to pulsotype P4 (strains 160U and 178U), 16.66% (5/30) belonged to pulsotype P5 (strains 339H, 385H, 581H, 635H, and 653H), 10.00% (3/30) belonged to pulsotype P6 (strains 695H, 717H, and 723H), and 40.00% (12/30) belonged to pulsotype P7 (strains 41H, 50H, 63H, 483H, 488H, 499H, 899H, Env1, Env2, Env6, Env7, and Env8; Figure 2). The pulsotypes P2, P3, and P7 of *S. maltophilia* strains showed a clonal relationship with 100% similarity among themselves; however, P4, P5, and P6 were associated only with clinical *S. maltophilia* strains (Figure 2).

**Figure 3:**
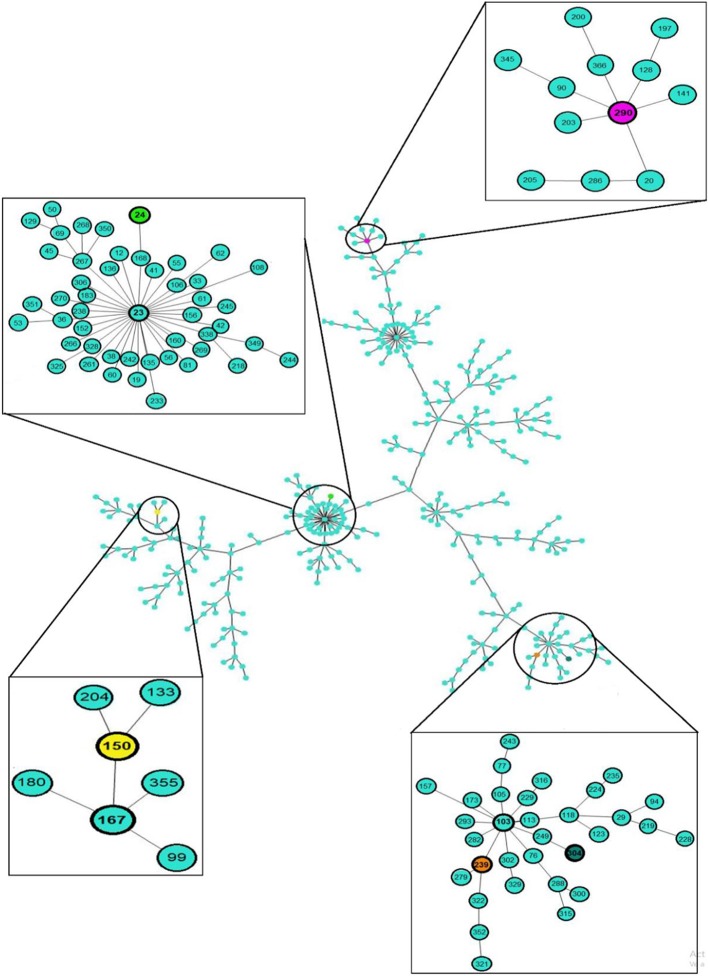
eBURST analysis of 30 clinical and environmental strains of *S. maltophilia*. The sequence type (ST) and clonal complex (CC) associated with the outbreak in the emergency department of the HIMFG are shown in boxes. The population distribution of the strains worldwide is also shown. Minimum spanning trees of the clinical and environmental strains of *S. maltophilia* were built using PHYLOViZ Online with the goeBURST algorithm, and this analysis considered 1,370 sequences reported in the MLST database.

The MLST analysis showed that the clinical and environmental strains of *S. maltophilia* were grouped into five STs exhibiting the following distributions: ST304 [50.0% (15/30)], ST239 [26.66% (8/30)], ST24 [13.33% (4/30)], ST150 [6.66% (2/30)], and ST290 [3.33% (1/30); Figures 2, 3, Table S3].

The environmental *S. maltophilia* strains were related to two STs: ST304 [77.77% (7/9)] and ST24 [22.22% (2/9)]. The *S. maltophilia* strains from blood samples were associated with three STs: ST304 [47.06% (8/17)], ST239 [41.17% (7/17)], and ST24 [11.76% (2/17)]. In addition, the *S. maltophilia* strains from urine samples were associated with three STs: ST150 [50% (2/4)], ST239 [25% (1/4)], and ST290 [25% (1/4)]. The allelic profiles of the clinical and environmental strains of *S. maltophilia* are shown in Table S3. The clonal complexes (CCs) identified in the *S. maltophilia* strains were CC103, CC23, CC167, and CC290 (Figure 3, Table S3).

### Resistance Profiles of the *S. maltophilia* Strains

The resistance profiles for six antibiotic categories were determined according to the CLSI (2018). *S. maltophilia* strains were considered MDR if they were not susceptible to at least one agent in three or more categories of antibiotics, whereas strains were considered pandrug-resistant (PDR) if they were not susceptible to all the agents in all the antibiotic categories (Magiorakos et al., 2012). Furthermore, *S. maltophilia* strains were considered resistant (R) if they were not susceptible to at least one agent in one to two antibiotic categories, and strains were considered susceptible if they were not resistant to all the antibiotic categories. According to the resistance profiles, 10.00% (3/30) of the *S. maltophilia* strains were PDR, 46.66% (14/30) were MDR, 33.33% (10/30) were R, and 10.00% (3/30) were susceptible to all the antibiotics tested. Moreover, 80.00% (24/30) of the *S. maltophilia* strains were resistant to TE, 76.66% (23/30) were resistant to SXT, and 23.33% (7/30) were producers of ESBL (Table 1).

**Figure 4:**
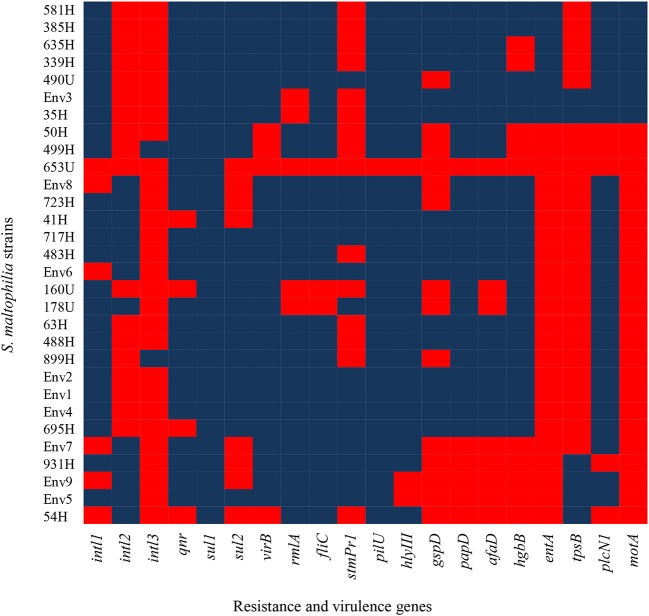
Heat map of putative virulence genes and resistance genes in the clinical and environmental strains of *S. maltophilia*. The blue and red colors indicate the presence and absence of a gene, respectively.

Class 1, 2, and 3 integrons were identified in the following proportion: 80.00% (24/30) of the *S. maltophilia* strains exhibited class 1 integrons, 40.00% (12/30) exhibited class 2 integrons, and 6.66% (2/30) exhibited class three integrons. Additionally, the other resistance genes of the *S. maltophilia* strains included in this study were distributed as follows: 86.66% (26/30) of the strains contained the *qnr* gene, 100% (30/30) contained the *sul1* gene, and 73.33% (22/30) contained the *sul2* gene (Figure 4).

### Frequency of Virulence Genes in the *S. maltophilia* Strains

The *S. maltophilia* strains related to virulence genes exhibited the following distribution: 86.6% (26/30) harbored the *virB* gene, 83.33% (25/30) harbored the *rmlA* gene, 90.0% (27/30) contained the *fliC* gene, 50.0% (15/30) contained the *stmPr1* gene, 96.6% (29/30) contained the *pilU* gene, 90.0% (27/30) contained the *hlyIII* gene, 53.3% (16/30) contained the *gspD* gene, 80.0% (24/30) contained the *papD* gene, 73.33% (22/30) contained the *afaD* gene, 66.66% (20/30) contained the *hgbB* gene, 23.33% (7/30) contained the *entA* gene, 20.0% (6/30) contained the *tpsB* gene, 83.33% (25/30) contained the *plcN1* gene, and 23.33% (7/30) contained the *motA* gene (Figure 4).

**Figure 5:**
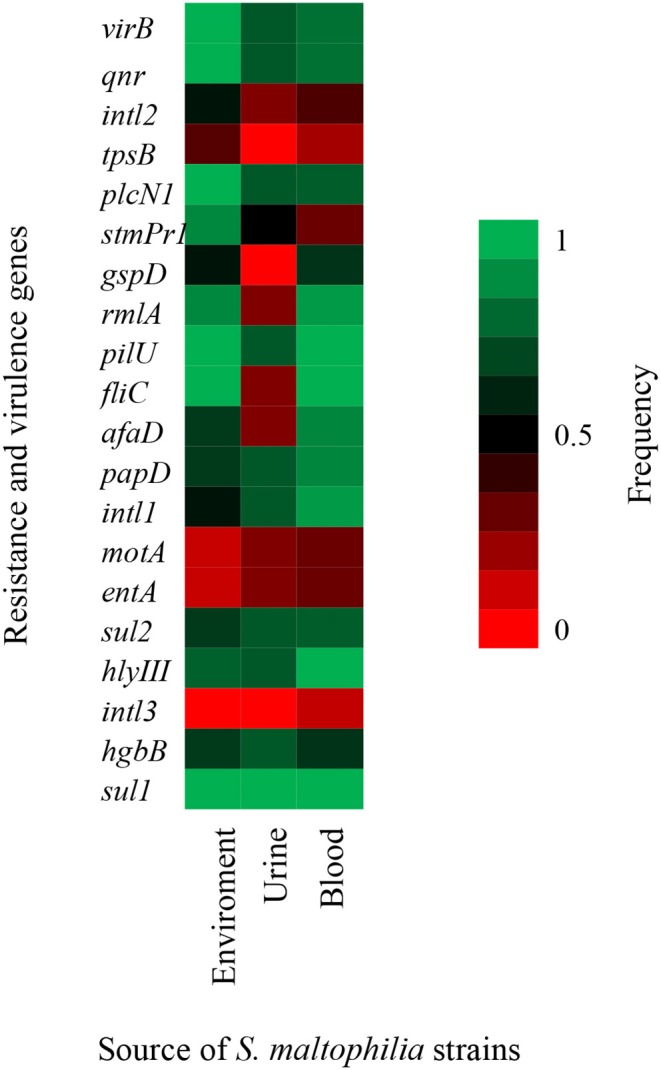
Heat map of putative virulence genes and resistance genes in nine strains of *S. maltophilia* from environmental sources, 17 strains from blood sources, and four strains from urinary sources. The percentage of strains harboring each gene is depicted by a color scale from red to green.

In addition, other virulence genes, such as *zot, fhaB, frpC, fimH, lktD*, and *hcp*, were not identified in the *S. maltophilia* strains (data not shown). By contrast, all environmental *S. maltophilia* strains 100% (9/9) contained the *virB, fliC, pilU, plcNI, qnr*, and *sul1* genes, all *S. maltophilia* strains that originated from blood samples showed 100% (17/17) positivity for four genes (*fliC, pilU, hlyIII*, and *sul1*), and 100% (4/4) from urine samples showed positivity for the *sul1* gene from urine samples (Figure 5).

### HTB-9 Cells Adhesion and Biofilm Formation Capacities of *S. maltophilia* Strains

**Figure 6:**
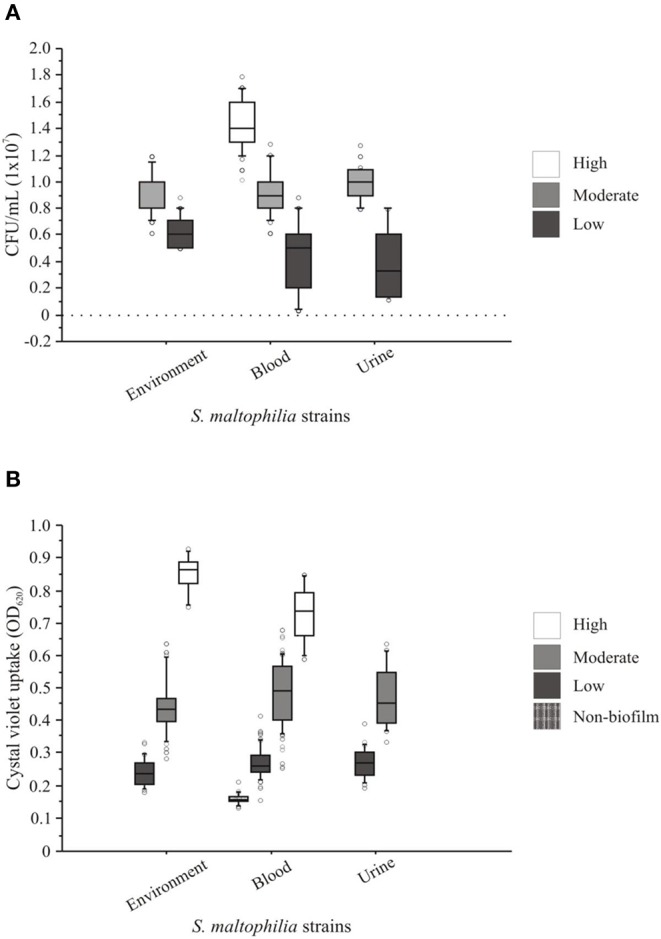
Adherence and biofilm patterns of clinical and environmental strains of *S. maltophilia*. **(A)** The adhesion assays were performed with human bladder HTB-9 cells. **(B)** The biofilm assays were based on the spectrophotometric quantification of the methanol retained by the 96-well microplate biofilm. The points outside the boxes represent the values of adhesion and biofilm formation outside the quartiles.

The attachment of *S. maltophilia* strains to monolayers of HTB-9 cells exhibited a heterogeneous profile of adherence with different values of CFU/mL. The adherence levels ranged from 0.04 × 10^7^ to 1.6 × 10^7^ CFU/mL (Figure 6A) and were classified as weakly <0.9 × 10^7^ CFU/mL), moderately (0.9–1.16 × 10^7^ CFU/mL), or strongly (≥1.16 × 10^7^ CFU/mL) adherent according to the mean value (1.05 × 10^7^ CFU/mL, SD ± 1.29) obtained for the positive control (*S. maltophilia* ATCC 13637). The analysis of the *S. maltophilia* strains from environmental sources revealed that 55.5% (5/9) were weakly adherent, 44.4% (4/9) were moderately adherent, and none were strongly adherent (Figure 6A). Among the *S. maltophilia* clinical strains originating from blood, 41.17% (7/17) were weakly adherent, 29.41% (5/17) were moderately adherent, and 29.41% (5/17) were strongly adherent. In addition, 50.0% (2/4) of the *S. maltophilia* clinical strains originating from urine were weakly adherent, and the others 50% (2/4) were moderately adherent. Interestingly, the *S. maltophilia* clinical strains showed increased adherence compared with the *S. maltophilia* strains from environmental sources (Figure 6A).

The *S. maltophilia* strains associated with the formation of biofilms showed a heterogeneous profile independent of their source of isolation. The strains were classified as follows: 10.0% (3/30) had a high biofilm-formation activity (>0.598 OD_590_), 46.66% (14/30) had a moderate biofilm-formation activity (>0.299 to ≤0.598 OD_590_), 36.66% (11/30) had a low biofilm-formation activity (>0.15 to ≤0.299 OD_590_), and 6.66% (2/30) did not form biofilms (≤0.15 OD_590_). In addition, 41.17% (7/17), 50% (2/2), and 55.55% (5/9) of the *S. maltophilia* strains originating from blood, urine, and the environment, respectively, showed moderate biofilm-formation activity (Figure 6B).

### Twitching and Swimming Motilities of the *S. maltophilia* Strains

**Figure 7:**
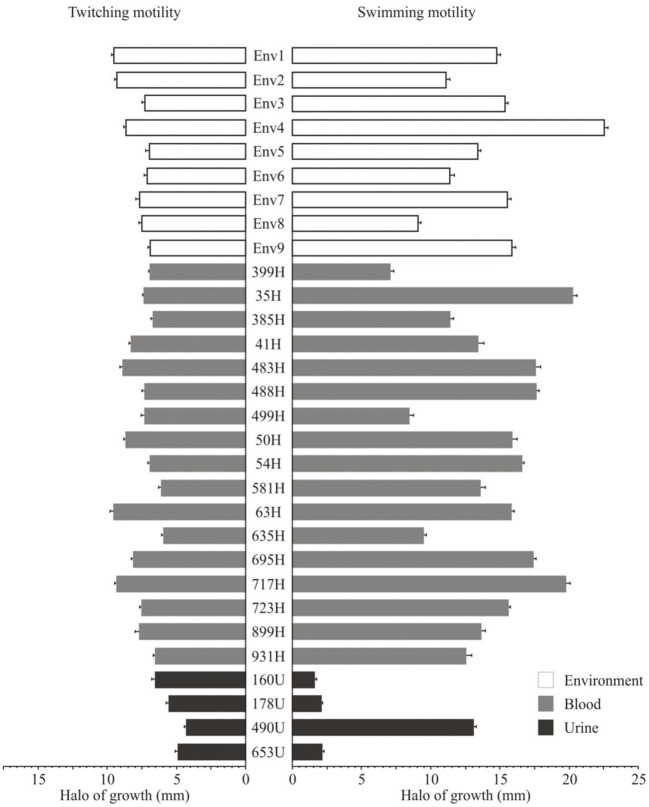
Twitching motility and swimming motility of the clinical and environmental strains of *S. maltophilia* associated with a hospital outbreak.

The twitching motility of the clinical and environmental strains of *S. maltophilia* showed heterogeneous profiles with different halo diameters: 13.33% (4/30) were classified as high (>9.0 mm/diameter), 43.33% (13/30) as moderate (>7.0 to ≤9.0 mm/diameter), 26.66% (8/30) as low (>6.0 to ≤7.0 mm/diameter), and 16.66% (5/30) did not show any twitching motility (≤6.0 mm/diameter) phenotype. Among the environmental *S. maltophilia* strains, 33.33% (3/9) exhibited low, 44.44% (4/9) showed moderate, and 22.22% (2/9) presented high twitching motility. In addition, 75% (3/4) of the *S. maltophilia* strains from urinary sources did not show any twitching phenotype. Interestingly, among the *S. maltophilia* strains from blood cultures, 11.76% (2/17) showed high, 52.94% (9/17) exhibited moderate, 23.52% (4/17) showed low, and 11.76% (2/7) presented no twitching motility (Figure 7).

The swimming motility of the *S. maltophilia* strains showed the following distribution: 76.66% (23/30) presented high (>10.0 mm/diameter), 10.0% (3/30) exhibited moderate (>7.0 to ≤10.0 mm/diameter), and 3.33% (1/30) exhibited low motility (>6.0 to ≤7.0 mm/diameter). The environmental and blood culture strains were motile with rates of 88.88% (8/9) and 82.85% (14/17), respectively; however, 75% (3/4) of the strains from urinary sources lacked the *fliC* gene and showed no motile phenotype (Figure 7).

### Association of *S. maltophilia* Strains Using Chord Graphs

**Figure 8:**
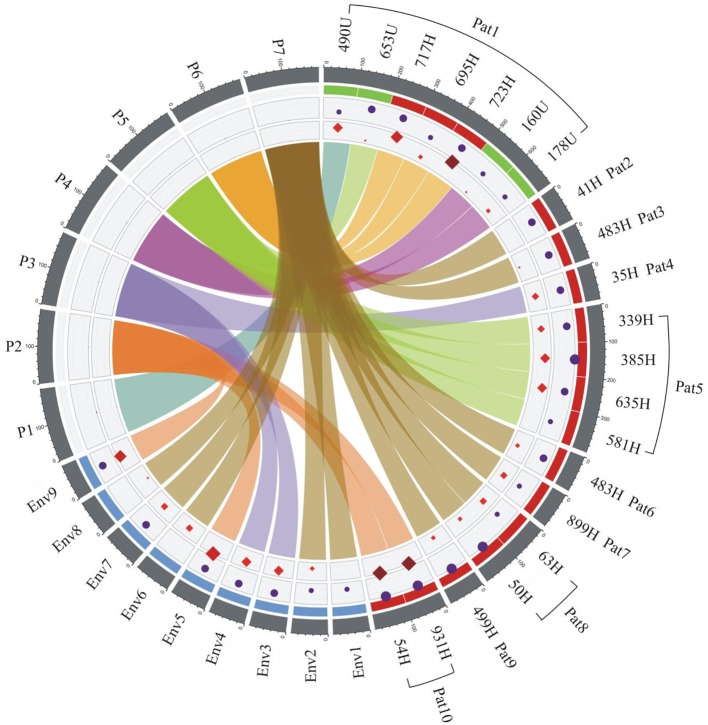
Epidemiological analysis of the clinical and environmental strains of *S. maltophilia* related to the outbreak. The associations among the source of isolation, pulsotypes (P1–P7), adherence, and resistance profiles of *S. maltophilia* strains were derived using ShinyCircos software. The outer ring shows the patient number (Pat1–Pat10) and source of strains: H, blood in red; U, urine in green; and Env1-9, environmental in blue. The inner rings are color coded: purple circles represent adherence, and the red diamonds illustrate the resistance profile of each strain. The size of the circles and diamonds is proportional to bacterial adherence (CFU/mL) and the number of antibiotic resistance genes, respectively. At the center, every pulsotype is associated with the clonal relatedness of the strain and is represented with a different chord color: P1 (turquoise), P2 (dark orange), P3 (blue), P4 (purple), P5 (green), P6 (light orange), and P7 (brown). The connected colors indicate the presence of a specific strain in either the patient samples or the environmental samples. The brown, purple, and dark orange colors indicate the faucet samples, and all three of these strains showed a connection with those from the patients: the strain represented in brown was associated with Pat2, 3, 6, 7, 8, and 9; the strains represented in purple were associated with Pat4; and the strains represented in dark orange were associated with Pat10. Pat1 and 5 were not associated with a faucet-sourced strain. Pat1 was associated with four different strains (turquoise, green, light orange, and purple), and one of these strains (green) was associated with Pat5. Env, environmental; Pat, patient. The number after Env or Pat indicates the number of the environmental or patient samples, which were numbered continuously.

The clinical data, resistance profiles, and adherence patterns and clonal relatedness of the clinical and environmental strains of *S. maltophilia* were visualized using chord diagram software. The analysis of these data indicated three main connections with a relationship among them (Figure 8). Environmental strains were associated with 80% (8/10) of the infected patients, and two patients (1 and 5) did not show a relationship with these strains. No association was observed between the urinary and environmental strains of *S. maltophilia*. The environmental strains (Env1, Env2, Env6, Env7, and Env8) showed a correlation with the blood-sourced strains in six patients, as indicated in a brown color, and the Env3 and Env4 strains were related to the blood sample from one patient. The Env5 and Env9 strains were related to two blood samples from one patient (Figure 8).

## Discussion

*S. maltophilia* is an aerobic, non-fermentative, gram-negative bacterium and an opportunistic pathogen that has been associated with serious infections in ICUs and nosocomial outbreaks (Gulcan et al., 2004; Guyot et al., 2013). The infection frequently occurs secondarily to the use of broad-spectrum antibiotics, prolonged hospitalization, ICU stays, mechanical ventilation, indwelling catheters and the use of equipment in contact with the respiratory tract (Pompilio et al., 2016). The HIMFG treats immunocompromised patients with diseases in specific organs, which increases the risk of opportunistic infections. In this study, we investigated an outbreak associated with *S. maltophilia* in the HIMFG and characterized the antibiotic resistance profiles, molecular epidemiology, and frequencies of virulence genes. *S. maltophilia* has been isolated from several nosocomial sources, such as blood-sampling tubes, ventilator circuits, dialysis machines, shower heads, sink traps, water faucets, and the hands of healthcare personnel (Denton and Kerr, 1998). *S. maltophilia* bloodstream infections are associated with the inappropriate use of antibiotics, long-term hospital stays, chronic diseases (including cancer), and immunocompromised patients (Wu et al., 2006; Chang et al., 2014).

*S. maltophilia* has high genetic diversity, as demonstrated with various typing methods (Gherardi et al., 2015; Pompilio et al., 2016; Alcaraz et al., 2018; Guvenir et al., 2018). However, in Mexico, 75–100% similarity has been obtained among strains collected from the same hospital source over a period of 7 years (Flores-Trevino et al., 2014). In this study, the PFGE and MLST analyses grouped the environmental strains of *S. maltophilia* into three pulsotypes and two STs. P3 (Env3 and Env4) and P7 (Env1, Env2, Env6, Env7, and Env8) were associated with ST304, whereas P2 (Env5 and Env9) was associated with ST24. These results indicate that PFGE remains the gold standard for DNA fingerprinting and exhibits substantial power for genetic and epidemiological discrimination in the evaluation of hospital outbreaks (Gherardi et al., 2015). However, MLST enabled identification of the clonal groups associated with the strain STs, which facilitates their monitoring worldwide. In addition, MLST provides information about the population structure as well as the long-term global and evolution history (Gherardi et al., 2015). For instance in our study, the *S. maltophilia* strains isolated from blood samples were associated with five pulsotypes and three STs. P2 was related to ST24, P3 was associated with ST304, P7 was related to ST304, P5 was related to ST239, and P6 (strains 695H, 717H, and 723H) was associated with ST239. The *S. maltophilia* strains isolated from urine samples were mainly associated with P4 and ST150. The clinical *S. maltophilia* strains were highly related and distributed as follows: P1 was related to ST290, P4 was related to ST150, P5 was related to ST239, and P6 was related to ST239. Furthermore, these clusters did not show a clonal relationship with *S. maltophilia* strains from environmental sources, which suggested an additional source of infection.

Interestingly, 47.61% (10/21) of the clinical *S*. *maltophilia* strains were clonally related to environmental strains obtained from faucets and spouts. Our data suggested that the environmental strains collected from faucets were identical to the clinical strains obtained from eight patients, which suggests a partial source of the outbreak. The HIMFG has as a policy of hand washing before taking any sample or performing any invasive procedure as well as before entering the emergency department (De la Rosa-Zamboni et al., 2018). A previous outbreak of *S. maltophilia* strains isolated from blood was associated with cross-contamination from the hands of healthcare workers (Park et al., 2008). The findings found in this study support the hypothesis that contamination by health workers is a probable source of infection and transmission among patients (De la Rosa-Zamboni et al., 2018). During the outbreak, the results obtained after the implementation of faucet disinfection and sterilization as a control measure indicated that cross-contamination is important for strain transmission in the emergency department.

Seven *S. maltophilia* strains with different infection processes were isolated from one patient. These strains were grouped into four pulsotypes (P1, P4, P5, and P6) and three STs (290, 150, and 239), which indicated the existence of a mixed infection in this patient. The urine-sourced *S. maltophilia* strain 490U included in P1 showed no relationship with other strains, which indicated that this strain might be found in different environmental sources possibly of community origin (Chang et al., 2014). The urine-sourced *S. maltophilia* strain 653U was grouped into P5 with four strains that were obtained from the blood of two other pediatric patients, which suggested the existence of cross-colonization in the patients with the same strains.

*S. maltophilia* strains have been associated with outbreaks in ICUs due to their MDR profiles, which enhance cross-nosocomial colonization (Gales et al., 2001; Sanchez, 2015). The MDR profiles of the *S. maltophilia* strains exhibit resistance to SXT and TE, which are recommended as the agents of choice for the treatment of this species (Flores-Trevino et al., 2014; Hu et al., 2016; Brooke et al., 2017). Interestingly, three *S. maltophilia* clinical strains (54H, 931H, and 178U) presented a PDR profile, which might be characteristic of circulation in hospital environments. In addition, multidrug resistance and resistance to stress are characteristics that facilitate the competence of *S. maltophilia* in areas with intensive microbial activity, such as the nosocomial environment (An and Berg, 2018). The *sul* and *dfrA* genes, class 1 integrons and mobile genetic elements contribute to SXT resistance (Chang et al., 2007; Hu et al., 2011). Moreover, resistance to SXT is associated with quaternary ammonium compounds (QACs); thus, the *qacE*Δ*1* gene is related to SXT resistance. Therefore, the use of disinfectants containing QACs might increase the risk of SXT resistance in hospital settings and natural environments (Gaze et al., 2005). Unfortunately, only the *sul1* and the *sul2* genes were included in this study. Further studies are needed to detect other genes, such as *aqE*Δ*1-sul1, dfrA12, dfrA17*, and *dfrA27*, which are involved in the wide and rapid dissemination of resistance genes in bacteria in other countries (Hu et al., 2016). It is important to comment that the integrase gene classes 1, 2, and 3 were used as indicators of the presence of integrons in this study. However, additional sequencing studies of the integron variable region are necessary for identification of the cassette genes associated with resistance in *S. maltophilia* strains. Interestingly, the *qnr* gene that encodes resistance to quinolones was the most important characteristic of environmental *S. maltophilia* strains, which suggests that multidrug resistance favors competition in ecological niches of several microorganisms (Malekan et al., 2017). The presence of *intl1* is indicative of the class 1 integron and was found in a high percentage of *S. maltophilia* strains originating from blood samples, which indicates that it could serve as a mechanism for facilitating the dissemination of resistance at the hospital level (Malekan et al., 2017).

The virulence genes of *S. maltophilia* strains from clinical and environmental sources have been described; however, the functions of other virulence genes have not yet been described, and these genes are considered putative virulence genes (Adamek et al., 2014). In this study, the gene profiles of the *S. maltophilia* strains from environmental and clinical sources showed high frequencies of virulence genes involved in swimming motility (≥80%), secretion systems (0–50%), toxins (20–60%), and siderophores (20%). The *pilU* (pili type 4) and *fliC* (flagellin) genes in the environmental and blood-sourced strains of *S. maltophilia* suggest an association with biofilm formation to facilitate their persistence in faucets. The *virB* gene (TSS4) was strongly associated with environmental strains, which suggests that the participation of secretion systems is essential for environmental prevalence. In addition, hemolysin III is a toxin that facilitates strain survival in the bloodstream (Adamek et al., 2014).

*S. maltophilia* strains have the ability to adhere to and form biofilms on medical devices, such as prosthetic devices and blood and urinary catheters, and have been associated with hospital outbreaks. Biofilms provide protection against antibiotics and immune system defenses (De Oliveira-Garcia et al., 2003). In this study, *S. maltophilia* strains from environmental and clinical sources showed inherent profiles of biofilm formation, which indicates that the biofilm-formation capacity is essential for bacterial persistence regardless of the colonization sites; indeed, only two blood-sourced strains did not form biofilms (Pompilio et al., 2008, 2011). The flagella of *S. maltophilia* strains are structural appendages that contribute to the appearance of hospital outbreaks (De Oliveira-Garcia et al., 2002). Our assessment of the swimming motility and the finding that 90% of the *S. maltophilia* strains that harbored the *fliC* gene, including the environmental and clinical strains, showed a heterogeneous profile reflecting a high swimming motility, which indicated that the flagella are required for the colonization of these strains in the host. Under these same conditions, 56.66% of all the *S. maltophilia* strains (43.33% of the blood-sourced strains and 13.33% of the urine-sourced strains) showed twitching motility. The strains from environmental and blood sources harbored the *pilU* gene, which is associated with the presence of the type 4 pili in *S. maltophilia*.

Adherence to epithelial cells is the initial step in the colonization or invasion of different epithelial cells, such as those in the respiratory and urinary tracts, injured skin and wounds, by *S. maltophilia*. Our results showed that the blood-sourced strains exhibited increased adherence to human bladder HTB-9 cells compared with the strains from environmental and urinary sources. The ability to adhere to plastic venous catheters and survive within intravenous solutions can contribute to the pathogenesis of bloodline-associated infections (De Oliveira-Garcia et al., 2003). Additionally, adherence to epithelial cells is related to the presence of fimbriae or pili that directly mediate the binding of bacteria to their target host cells or indirectly mediate binding by the formation of cross-junctions between cells (De Oliveira-Garcia et al., 2003). Thus, the typed genotypes of the *S. maltophilia* strains isolated from blood samples indicated the presence of a high percentage of genes associated with colonization, such as *papD, afaD, pilU*, and *fliC*, which is in agreement with the literature. The finding that the *S. maltophilia* strains of blood origin adhered to HTB-9 cells indicates their participation in the permanence and dissemination of strains in the hospital environment.

Finally, the outbreak was controlled through the routine disinfection of faucets and spouts. The policies for the routine or terminal disinfection of a clinical ward do not usually include disinfection of the internal faucet parts (Rutala and Weber, 2008; Chou, 2014). In an emergency department, water splashes generated between spouts and faucets can contaminate the internal part of faucets that harbor environmental bacteria with the ability to form biofilms, which is favored by several virulence factors. The emergency department of the HIMFG requires renovation and structural optimization, including the installation of sinks with adequate dimensions, to avoid bacterial persistence in areas at a high risk of contamination, and these measures are ongoing. In conclusion, the outbreak associated with *S. maltophilia* in the HIMFG was partially related to faucet contamination in the emergency department. Other pulsotypes and STs (150, 239, and 290) were not found in the environmental strains of *S. maltophilia*, which suggests other infection sources. Their clonality, selected virulence genes, and resistance mechanisms are attributes associated with the success of infection and persistence by these strains in the hospital and facilitated the outbreak. Finally, these findings support the need for the implementation of disinfection policies that include the disinfection of faucets as a necessary strategy for the control of outbreaks in clinical areas.

## Data Availability Statement

All datasets generated for this study are included in the article/Supplementary Material.

## Ethics Statement

The projects with the registration numbers HIM/2019/067, HIM/2018/045FF SSA.1503, HIM/2018/049FF SSA.1504, and HIM/2016/099 SSA.1329 were submitted, reviewed, and approved by the Research Committee (Dr. Juan Garduño Espinosa, Research Director), Ethics Committee (Dr. Luis Jasso Gutiérrez, President of the Research Ethics Committee), and Biosecurity Committee (Dr. Marcela Salazar García, Leader of Biomedical Research Service and Research Biosecurity Committee) of the Children's Hospital of México Federico Gómez (HIMFG). The clinical *S. maltophilia* strains included in this study were obtained from blood and urine samples using routine clinical diagnostic procedures. Signed informed consent was provided at the time of sampling in the Central Clinical Laboratory of the Hospital. The informed consent form mentions that the sample might be used for research purposes according to the following reference: General Health Law chap. IV. Art. 80, 81, 82, and 83. NOM-007-SSA3-2011. México.

## Author Contributions

SO and JX-C designed and conceived the experiments, wrote and reviewed the manuscript. SO, GE-V, VC-D, and JM-R performed the experiments. SO, SZ-V, VL-P, AC-C, MM-P, IP-O, DR-Z, and JX-C analyzed the data. SO, SZ-V, AC-C, DR-Z, MH, CO, IF-H, and JX-C contributed reagents/materials/analytical tools.

### Conflict of Interest

The authors declare that the research was conducted in the absence of any commercial or financial relationships that could be construed as a potential conflict of interest.
