# Supplementary material for: Molecular Epidemiology, Antibiotic Resistance, and Virulence Traits of Stenotrophomonas maltophilia Strains Associated With an Outbreak in a Mexican Tertiary Care Hospital
Source: Front Cell Infect Microbiol. 2020 Feb 18;10:50. doi: 10.3389/fcimb.2020.00050 (PMC7040173; doi:10.3389/fcimb.2020.00050)
Supplement: Supplementary file 1 [file Table_1.docx]

**Table S1**. Specific primers used for the amplification of resistance genes and MLST in clinical and environmental *S. maltophilia* strains.

| **Gene** | **Primer sequences 5’-3’** | **Amplified fragment (bp)** | **Description** | **Reference** |
| --- | --- | --- | --- | --- |
| **Primers for amplification of resistance genes** | | | | |
| *qnr* | Smqnr-F: ACACAGAACGGCTGGACTGC  Smqnr-R: TTCAACGACGTGGAGCTGT | 817 | Quinolone resistance protein | Malekan et al., 2017 |
| *intl1^a^* | SMintl-1F: GGTCAAGGATCTGGATT  TGG  SMintl-1R ACATGCGTGTAAATCATC  GTC | 500 | Integrasa of integron class 1 | Ozkaya et al., 2014 |
| *intl2^a^* | SMintl-2F CACGGATATGCGACAAAAA  GGT  SMintl-2R GTAGCAAACGAGTGACGAA  ATG | 740 | Integrasa of integron class 2 | Ozkaya et al., 2014 |
| *intl3^a^* | SMintl-3F AGTGGGTGGCGAATGAGTG SMinti-3F TGTTCTTGTATCGGCAGGTG | 1166 | Integrasa of integron class 3 | Hu et al., 2016 |
| *sul1* | sul1-F TAGCGAGGGCTTTACTAAGC  sul1-R ATTCAGAATGCCGAACACCG | 921 | Dihydropteroate synthase type I | Hu et al., 2016 |
| *sul2* | sul2-F CCTGTTTCGTCCGACACAGA  sul2-R GAAGCGCAGCCGCAATTCAT | 437 | Dihydropteroate synthase type I | Hu et al., 2016 |
| **Primers for amplification of MLST** | | | | |
| *atpD* | atpD-F: ATGAGTCAGGGCAAGATCGTTC  atpD-R: TCCTGCAGGACGCCCATTTC | 858 | H(+)-transporting two-sector ATPase | Kaiser et al., 2009 |
| *gapA* | gapA-F: GCATCGGGCGTAACGTCCTGC  gapA-R: CTTCGCTCTGTGCCTTCACTTC | 768 | NAD-dependent glyceraldehyde-3-phosphate dehydrogenase | This study |
| *guaA* | guaA-F: AACGAAGAAAAGCGCTGGTA  guaA-R: ACGGATGGCGGTAGACCAT | 704 | GMP synthase [glutamine-hydrolyzing] | Kaiser et al., 2009 |
| *mutM* | mutM-F: ATGCCTGAACTGCCCGAAG  TCG  mutM-R: GCTGGTAGCGTTCGCGCGAG | 592 | DNA-formamidopyrimidine glycosylase | This study |
| *nuoD* | nuoD-F: GCGCGTGATCTACGAACCGGT  nuoD-R: GAAGTTCTCCAGGAAGTCCAG  CAG | 531 | NADH dehydrogenase [ubiquinone] | This study |
| *ppsA* | ppsA-F: GCTGTTCACCCTGGACACCGA  ppsA-R: CGAAGTCGAAGGCACGTT | 887 | Pyruvate, water dikinase | This study |
| *recA* | recA-F: ATGGACGAGAACAAGAAGCGC  recA-R: GGTGATGACCTGCTTGAACG | 807 | RecA protein | Kaiser et al., 2009 |

^a^ Only genes for the specific integrase were included, not for the integron variable region.
