# Supplementary material for: Molecular Epidemiology, Antibiotic Resistance, and Virulence Traits of Stenotrophomonas maltophilia Strains Associated With an Outbreak in a Mexican Tertiary Care Hospital
Source: Front Cell Infect Microbiol. 2020 Feb 18;10:50. doi: 10.3389/fcimb.2020.00050 (PMC7040173; doi:10.3389/fcimb.2020.00050)
Supplement: Supplementary file 2 [file Table_2.docx]

**Table S2**. Specific primers used for the amplification of virulence genes in clinical and environmental *S. maltophilia* strains.

| **Gene** | **Primer sequences 5’-3’** | **Amplified fragment (bp)** | **PCR** | **Reference** |
| --- | --- | --- | --- | --- |
| *lktD* | lktD-F: GCACATCCGTGATGCAGTCG  lktD-R: CGAGATTCTCGTCCTGCATGG | 1235 | Type I secretion system protein (T1SS) | This study |
| *gspD* | gspD-F: GTCGACACCGATATCGGTGG  gspD-R: GGTAGACCACATGCAGGTTGC | 694 | Type II secretion system protein (T2SS) | This study |
| *virB* | virB-F: GCATCATGCAGAACGAGCTG  virB-R: GACGGCTCGTACTTCTGCAC | 1075 | Type IV secretion system protein (T4SS) | This study |
| *hcp* | hcp-F: GACGGCAACGCGATCAATTAC  hcp-R: GTTCTTGGTTGCACTCCACTG | 201 | Type VI secretion system protein (T6SS) | This study |
| *entA* | entA-F: CGTTCGCACTCGACGTGAC  entA-R: CGAACTGACGGTAACGATCACG | 251 | Putative enterobactin synthetase component A | This study |
| *tpsB* | tpsB-F: GTGGACATCGTGATGAAGCGC  tpsB-R: CTTGCCGATGAAGTGACGGTG | 822 | Channel-forming transporter/cytolysins activator (T5SS) | This study |
| *plcN1* | plcN1-F: GTGACCGATATCGGCCGAC  plcN1-R: CTGGAAGTGGCGGTGGAAG | 1779 | Putative non-hemolytic phospholipase C | This study |
| *stmPr1* | StmPr1-F: TGAAAGCAAATGCGCCGTTG  StmPr1-R: GTGATGGCGTCGGTGATGTC | 852 | Alcaline-serine-protease | This study |
| *rmlA* | rmlA-F: CTCAGCGTGCTGATGCTGG  rmlA-R: GATGAAGTTGGAGGCTTCC  AGC | 600 | Glucose-1-phosphate thymidylyltransferase (LPS) | This study |
| *hlyIII* | hly-F: CGTCCATTGCTTCGATCCGTG  hly-R: GACGAAGTGGCAGACGCTG | 607 | Putative transmembrane hemolysin protein III | This study |
| *hgbB* | hgbB-F: GGACATCCAGAACATGGGTGC  hgbB-R: GGATCGATCGTGTACGGACC | 1239 | Hemoglobin binding protein | This study |
| *zot* | Zot-F: GCGTCAGTACACCGATGGTTG  Zot-R: GCAGGCAGTGTCCAGCATG | 431 | Zot-toxin | This study |
| *frpC* | frpC-F: CCAGTTCAACCTGTCGATGCTG  frpC-R: CACCGAACAGGTTGTCCCAG | 653 | REpeats-in-toxin (RTX) toxin | This study |
| *pilU* | pilU-F: CGACCACCATCGATTTCACTTCG  pilU-R: GACAGGTCCATCAGCAGCTG | 778 | Putative PilU protein (Twitching motility protein) | This study |
| *fliC* | fliC-F: CGATCTCCGAGCGCTTCG  fliC-R: GAACAGCTGGCTGGAGAACG | 296 | Flagellin | This study |
| *afaD* | afaD-F: GAAGCGCCTGACTGCCTTTTG  afaD-R: GATCACGTTGTAAGGCCGCC | 328 | Putative AfaD non-fimbrial adhesin | This study |
| *papD* | papD-F: CACGCGAGTGATCTATCCGG  papD-R: GTGATGAAGCGCACCTGGTC | 579 | Putative pili chaperone protein | This study |
| *motA* | motA-F: CGTTGGATTCCTGGTCGTCATC  motA-R: GAGCCCATGGTGATGACGATG | 558 | Flagellar motor protein | This study |
| *fhaB* | fhaB-F: GTATCGCACAACCGCTTCCAG  fhaB-R: CGTCGTTGATGACCTTCTGCAC | 1744 | Hemmaglutinin | This study |
| *fimH* | fimH-F: GATCCGCCTGAACTGCCAG  fimH-R: CTGGCAGTTCAGGCGGATC | 576 | Putative adhesin of fimbria type1 | This study |
