# Supplementary material for: Molecular Epidemiology, Antibiotic Resistance, and Virulence Traits of Stenotrophomonas maltophilia Strains Associated With an Outbreak in a Mexican Tertiary Care Hospital
Source: Front Cell Infect Microbiol. 2020 Feb 18;10:50. doi: 10.3389/fcimb.2020.00050 (PMC7040173; doi:10.3389/fcimb.2020.00050)
Supplement: Supplementary file 3 [file Table_3.docx]

**Table S3**. Allelic profiles, sequence type, and clonal complexes associated with clinical and environmental strains of *S. maltophilia*.

| **Strains** | **Allelic profiles** | | | | | | | **ST** | **CC** |
| --- | --- | --- | --- | --- | --- | --- | --- | --- | --- |
|  | ***atpD*** | ***gapA*** | ***guaA*** | ***mutM*** | ***nuoD*** | ***ppsA*** | ***recA*** |  |  |
| 490U | 16 | 80 | 210 | 55 | 20 | 129 | 72 | 290 | 290 |
| 54H, 931H, Env5, Env9 | 9 | 21 | 28 | 109 | 15 | 18 | 113 | 24 | 23 |
| 35H, 41H, 50H, 63H, 483H, 488H, 499H, 899H, Env1, Env2, Env3, Env4, Env6, Env7, Env8 | 93 | 65 | 232 | 130 | 63 | 168 | 130 | 304 | 103 |
| 160U, 178U | 85 | 85 | 119 | 77 | 4 | 103 | 75 | 150 | 167 |
| 339H, 385H, 581H, 635U, 653H, 695H, 717H, 723H | 93 | 122 | 180 | 45 | 63 | 69 | 5 | 239 | 103 |

*atpD* (H(+)-transporting two-sector ATPase), *gapA* (NAD-dependent glyceraldehyde-3-phosphate dehydrogenase), *guaA* (GMP synthase [glutamine-hydrolyzing]), *mutM* (DNA-formamidopyrimidine glycosylase), *nuoD* (NADH dehydrogenase [ubiquinone]), *ppsA* (pyruvate, water dikinase), *recA* (RecA protein), sequence type (ST), and clonal complex (CC), Env1-9 (environmental *S. maltophilia* strains), H (blood), U (urine).
